# A New Quantitative Method for the Non-Invasive Documentation of Morphological Damage in Paintings Using RTI Surface Normals

**DOI:** 10.3390/s140712271

**Published:** 2014-07-09

**Authors:** Marcello Manfredi, Greg Bearman, Greg Williamson, Dale Kronkright, Eric Doehne, Megan Jacobs, Emilio Marengo

**Affiliations:** 1 Department of Sciences and Technological Innovation, University of Easter Piedmont, Viale T. Michel 11, Alessandria 15121, Italy; E-Mail: emilio.marengo@unipmn.it; 2 ANE Image, Pasadena, CA 91104, USA; E-Mail: gregb@snapshotspectra.com; 3 Media Arts Faculty, New Mexico Highland University, Las Vegas, NM 87701, USA; E-Mails:acousticguru@gmail.com (G.W.); mejacobs@nmhu.edu (M.J.); 4 Georgia O'Keeffe Museum, Santa Fe, NM 87501, USA; E-Mail: conservator@okeeffemuseum.org; 5 Conservation Sciences, Pasadena, CA 91104, USA; E-Mail: eric@conservationsciences.org

**Keywords:** damage detection, reflectance transformation imaging, monitoring conservation, cultural heritage, 3D surface, change over time

## Abstract

In this paper we propose a reliable surface imaging method for the non-invasive detection of morphological changes in paintings. Usually, the evaluation and quantification of changes and defects results mostly from an optical and subjective assessment, through the comparison of the previous and subsequent state of conservation and by means of condition reports. Using quantitative Reflectance Transformation Imaging (RTI) we obtain detailed information on the geometry and morphology of the painting surface with a fast, precise and non-invasive method. Accurate and quantitative measurements of deterioration were acquired after the painting experienced artificial damage. Morphological changes were documented using normal vector images while the intensity map succeeded in highlighting, quantifying and describing the physical changes. We estimate that the technique can detect a morphological damage slightly smaller than 0.3 mm, which would be difficult to detect with the eye, considering the painting size. This non-invasive tool could be very useful, for example, to examine paintings and artwork before they travel on loan or during a restoration. The method lends itself to automated analysis of large images and datasets. Quantitative RTI thus eases the transition of extending human vision into the realm of measuring change over time.

## 1. Introduction

The conservation, preservation, and documentation of cultural heritage have concerned conservators, art historians, and scientists for many years. The conservation of an artwork depends on the materials of which it is composed and on complex processes such as aging and environmental effects. The accuracy of monitoring techniques to evaluate these changes is indispensable for proper conservation and restoration strategies.

Paintings consist of different layers of materials. These generally consist in a support, primer, and layers of paint and varnish. Other layers such as glue, a preparatory drawing, an imprimatura and additional layers of paint can complement the painting composition. Moreover, the composition of paintings is often dominated by composite materials with different behaviors and chemistry. In general, damage can be defined as an adverse change from an original state.

Deterioration of paintings can be caused by environmental factors such as fluctuations in temperature and relative humidity, light radiation, and microbiological activity. In addition transportation can be another source of damage: vibrations, environmental climate changes, and poor handling can result in damage. The cumulative effects of these factors produce changes in both the painting structure and its chemical composition [1,2].

In traditional conservation techniques the evaluation and quantification of small changes and defects results mostly from an optical and subjective assessment, through the comparison of the previous and the subsequent state of conservation and by means of condition reports. A challenge for conservators is to distinguish a new defect from an old one or to detect the expansion of a defect when it is still in an early state of change.

Burmester *et al.*, Hein *et al.*, Kalms *et al.*, and Groves *et al.* [3–6] applied various optical methods to. For the first time a quantitative multispectral imaging technique coupled to statistics, developed by our group, has been used for the non-invasive monitoring of the conservation state of the Dead Sea Scrolls [7,8], one of the most important archaeological discoveries of the 20th century.

Other approaches like 3D scanning and shearography present advantages for the non-invasive analysis of artwork. Bertesaghi *et al.*, and Wegner *et al.*, and Debevec *et al.*, extracted the 3D information in the form of surface normals by collecting multiple images under varying lighting conditions: this information has been used for enhancing surface details [9–11].

Several paintings have been examined, showing that RTI is able to record surface features including craquelures, planar distortions, wood grains and canvas weaves. RTI renderings made before and after physical changes to a painting were compared and showed how promising the technique is for the examination of alterations in texture and shape of paintings [12].

Reflectance Transformation Imaging (RTI) is an imaging method that uses multiple angles of illumination to generate topographical information on the surface being imaged. RTI generates surface reflectance information using photometric stereo. The most common implementation of RTI is via Polynomial Texture Mapping (PTM) invented by Tom Malzbender of HP Labs in 2000 [13,14]. Alternative approaches were identified to compress defined surface properties including spherical and hemispherical harmonics [15]. RTI methods have been applied to archaeological and fine art objects in a wide variety of situations [16–18].

The theory of statistical process control (SPC) [19–22] was used to investigate superficial degradation processes, as has been previously demonstrated by our group in monitoring the conservation state of wooden objects and canvas painted with inorganic pigments, analyzed by Raman and IR spectroscopy [23,24].

In this paper we propose a reliable RTI method for the non-invasive detection of morphological changes in paintings: the method includes an imaging protocol, the extraction of the surface normal, and comparison with statistical limits derived from SPC theory. In order to have a reproducible and quantitative method that can be used as a practical method for measuring change in objects we developed a custom semi-arch arm with LED lights to illuminate the surface of a painting. Our method is a completely non-invasive monitoring technique: we obtained a quantitative measure of the deterioration and degradation present in the painting after inducing artificial damage.

## 2. Theory

### 2.1. Reflectance Transformation Imaging (RTI)

Malzbender *et al.* introduced a novel image-based relighting technique for visualizing the appearance of a surface under a spatially variable source of illumination. RTI is an image-based recording method in which information about the surface reflectance is captured on a per-pixel basis. RTI files are created from information derived from multiple digital photographs of an object shot from a stationary camera position: in each image light is projected from a different direction. This process produces a series of images of the same subject with varying highlights and shadows. Lighting information from the images is mathematically treated to generate a mathematical model of the surface, in the form of surface normals for each pixel, and used for enhancing various details [13–15]. A surface normal is a vector perpendicular to the surface at any given pixel. We will focus on that for measuring morphological changes to the surface [25]. An RTI file consists of an interactive viewing file where the user controls the angle of illumination of the surface imaged.

### 2.2. Quantitative RTI

Recently, the need to improve quantitative imaging techniques for monitoring purposes has become increasingly relevant. Our research is focused on the application of imaging methods for monitoring changes in cultural heritage objects. Quantitative imaging includes the development, standardization, and optimization of imaging acquisition protocols, data analysis, and results interpretation in order to have a validated, reliable, and precise method. This work is taking place within the larger field of computational photography and the measurement of the impact of environmental change on World Heritage.

### 2.3. Generating Surface Normal

Polynomial texture map (PTM) [14] and Hemispherical Harmonics (HSH) [26] function are usually used to describe the reflectance function. In general HSH approach is superior in terms of 3D quality because PTM uses only six coefficients of a biquadratic polynomial to describe the surface normal while a third order HSH uses 16 coefficients.

We have demonstrated that computing surface normals by means of the HSH polynomial function is more reproducible than using the older PTM method, and it is therefore much more sensitive to changes [25].

After capturing all images for RTI, each with a different light position and the same camera position, all the pictures, and respective light direction, for each single pixel are collected from all the images and fitted to the Hemispherical Harmonics function.

We used the open source software RTI Builder from Cultural Heritage Imaging (culturalheritageimaging.org) to build the RTI images from the raw image set and then calculated the normals for each pixel separately. Surface normals were extracted from RTI; the sixteen coefficients stored for each pixel in the HSH already contain the directional luminance information [26,27].

### 2.4. Change Detection Principles

Change measurement requires quantitative imaging methods: measuring change requires a before and an after image, quantitated well enough, with low noise, to perform useful comparisons.

In a previous work we have shown that RTI can be used as a sensitive monitoring methodology to automatically recognize areas that have changed: we evaluated how reproducible and accurate the method can be [25]. A monitoring method that has generally low reproducibility and accuracy (film photography, for example) means that it has poor sensitivity to change, since differentiating image changes from the image measurement uncertainty may not be possible.

Statistical process control is widely used in industrial applications and it has also been applied to cultural heritage monitoring. The general principle is to consider the conservation state as an industrial process, which is in its “in-control” condition when no deterioration effect is acting. Each further deviation from this initial condition is attributable to a damaging effect acting on the art object. The basic idea is that by monitoring the surface normals over time it is possible to detect the small-scale morphological and physical changes of an object surface. These changes affect the interaction of light with the object.

In this research we described the normal representing the vector using cartesian coordinates *x*, *y*, and *z*: the individual vector components were used as scalars and were statistically compared before and after the damages. In this way the results can be easily represented as maps, one for each coordinate, where pixels that are statistically changed are “painted” with a defined color.

A normal can be considered changed only if the *after* image value exceeds the region of statistical control which is defined by 3σ around the average value of each pixel, σ being the standard deviation of the considered pixel from the *before*, or control image. The average value and the standard deviation of each normal is calculated on its coordinates. This region corresponds to a confidence level of more than 99%. The average normal of each pixel and the region of statistical control have to be estimated by recording several replicates of RTI of the monitored object and are directly related to the uncertainty of the imaging technique: this baseline of normals set the timeline to zero and constitutes the characterization of the object. In this phase the object can be considered “in statistical control”. In this research the variance of the instrument, and so its reproducibility, has been assessed by recording five RTI replicates of the considered painting.

The absolute sensitivity of the method is the minimum variation of the normal that can be considered a statistically relevant change of the object: the larger the variance of the baseline normals, the less sensitive the method is.

**Figure 1.**
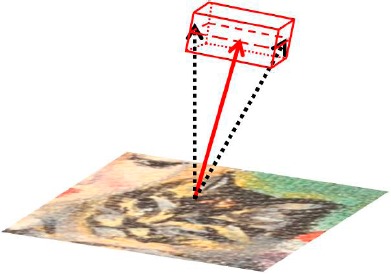
Pyramid with parallelepiped base that represents the region (volume) of the statistical limit within which a normal can be considered not changed.

In the present article the region of joint probability of *x*, *y* and *z* (coordinate of the surface normal vector) corresponding to the parallelepiped volume defined by the multivariate Gaussian probability function was approximated by the volume reported in Figure 1: this approximation is sufficiently conservative to provide reliable results. A normal is considered changed only if the monitored vector is outside the control volume.

## 3. Experimental Section

### 3.1. RTI Capture Technique

A D3100 camera (Nikon, Tokyo, Japan) with an 18 mm macro lens was used. The F-stop was set at f/8 and the exposure time at 0.62 s: both F-stop and the exposure time were fixed for all the acquisitions. The images are made by 4608 × 3072 pixels, the resolution of the images is 254 dpi and the ground pixel size is 0.1 mm. The camera was operated tethered to a PC.

The photographic capture of 48–52 digital images takes 8 min. It takes an additional 12 min to process and assemble the HSH-RTI data and image. This averages 20 min for a subject 1 m × 1 m or less. The greater the number of capture images, the longer the time to process and assemble.

### 3.2. Light

**Figure 2.**
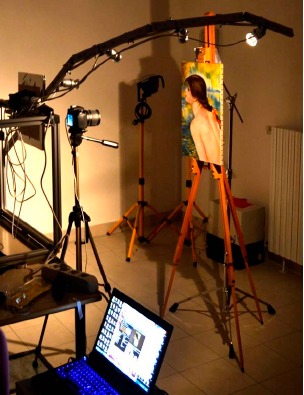
Instrument setup: semi arc arm with LEDs and RGB camera working on a painting.

A semi arch arm with four LEDs (20 Watt) at 15°, 30°, 45° and 60° lighting angles (Figure 2) was used to illuminate the surface of the painting to produce the highlight point in the reflective target. The object was illuminated from multiple light positions moving the arm every 20 degrees following a pattern with radial spokes, creating a virtual dome. The power of the lights was set to illuminate the surface of the object enough and was the same for each lamp. The lights were located from the surface of the object at a consistent distance of three times the object's diameter. A black reflective sphere was used to locate the lighting angle in each photograph [15]. The RTI Builder is able to identify automatically the light direction from its highlight on the sphere.

### 3.3. Image Processing

In this monitoring method the image alignment between imaging datasets is critical because mis-aligned pixels between imaging sessions would show up as topographical changes. Since it is not possible to repeatedly replace and image an object on the spatial scale of a pixel, rigorous digital alignment methods were employed.

The repeatability of the method was measured by recording the data in five separate imaging sessions: for every imaging session the camera and the object were repositioned in the same position. Then, the open source software RTI Builder was used to build the RTI images from the raw image set and the normals were extracted as described by Macdonald *et al.*, and Gautron *et al.* [24,26], using both Python version 2.7.3 and Matlab R2010a (the MathWorks, Natick, MA, USA).

The extracted normals of each replicate can be represented as three bi-dimensional images (one for each Cartesian coordinates *x*, *y*, *z*). In order to elaborate and compare the object over time, as described later in the paper, the images of *x*, *y*, and *z* must be aligned between each other. An open source image processing package, a version of ImageJ, called FIJI was used [28,29] to match the coordinate images of the five replicates. The color images, captured to build the RTI files, were employed to calculate an elastic transformation function (SIFT and bunwarpj tools were used) that was then used to align the *x*, *y*, and *z* coordinate images. In this way it was possible to align the images collected during different imaging sessions without positioning the painting in the same place. Moreover, using the elastic transformation function, which was based only on the color images and on their features, the method was able to align the coordinates images taken after a damage: in this case, the alignment has been performed without influencing the damaged areas because the information related to the normals is not accounted by the raw color images used to obtain the elastic function.

## 4. Results and Discussion

### 4.1. Painting

**Figure 3.**
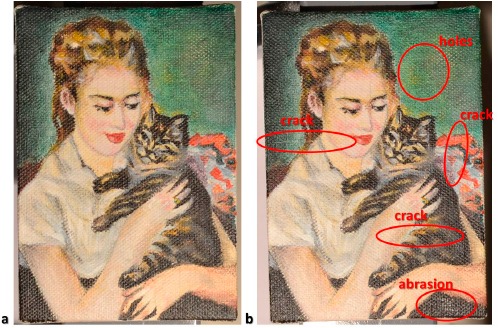
Color images of the painting before (**a**) and after (**b**) the artificial damage.

A painting on canvas of 10 cm × 7 cm was prepared and used for this research (Figure 3a). The canvas was painted with acrylic colors. The repeatability of the computed surface normals was measured by recording the data in five separate imaging sessions. The normals were computed with the HSH algorithm and the average variability was calculated. The surface normal vector is described representing the Cartesian coordinates *x*, *y*, and *z* of the vector itself: the average variability is calculated as standard deviation/mean × 100 for each pixel. The average measured variability was 5.8%, 6.9% and 3.8% respectively for *x*, *y*, and *z* axes. The variability of the measurement is very important because it is strictly correlated to the sensitivity of the method, namely the minimum variation that can highlight a statistically relevant change of the object. In the present case the repeatability was worse than the one we calculated in our previous work [27] even if a semi-arc arm was used with the aim of reducing the variability of the light positions and of saving time during each imaging sessions. This is likely due to the fact that in our previous work we used a DSLR camera that has more pixels (24 MP *versus* 14 MP) and hence a smaller IFOV sample size than the camera used for the present work.

### 4.2. Damage Detection in Painting

The five RTI replicates recorded with the aim of estimating the repeatability were used to characterize the system's “natural” variability whose estimation is necessary to build the control charts. The statistical limits were calculated as 3σ around the average value of each pixel and were used to define the interval containing the normals that did not present a statistical change as a consequence of the artificial deterioration processes applied. Figure 3 shows the color image of the painting used for this research. The painting was artificially damaged, after the characterization of the natural variability (Figure 3a), by creating some small holes, three cracks and one abrasion (red circles, Figure 3b).

After the damage, the RTI was captured again, and the surface normals were calculated. After aligning the images before and after damage, by comparing on a pixel basis these normals with the statistical limits calculated from the characterization images, it was possible to build a map of statistically significant changes (1% significance).

**Figure 4.**
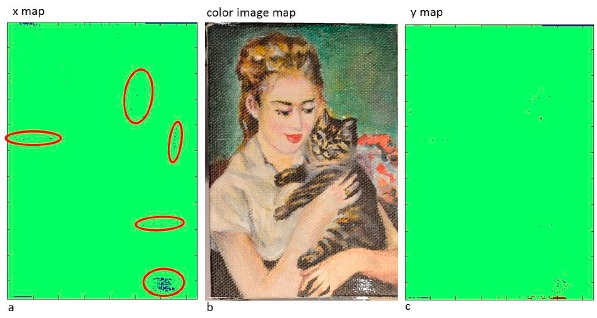
The map of changes for the *x* (**a**) and *y* (**c**) values of the normal: blue pixels represent the damaged areas. In (**b**) the color image map of change is represented: white pixels correspond to the damaged areas identified by the method.

Figure 4 shows three different ways to visualize the results: Figure 4a shows the map of changes for the *x* values of the normal (blue pixels represent the areas where the normals are characterized by statistically relevant changes); the map of changes for the *y* values of the normal is represented in Figure 4c. Figure 4b shows the color image of the painting where white pixels represent the damaged area identified by the method.

The maps of changes detected all the damages (red circles) that had been artificially made: so the method proved to be able to identify the changed areas.

**Figure 5.**
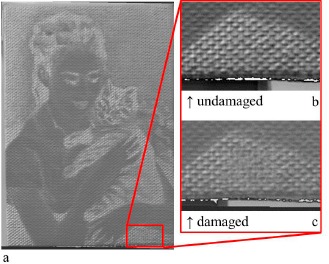
Bi-dimensional image of the *y* values of the normal of the damaged painting (**a**); magnification of a damaged area before (**b**) and after (**c**) the abrasion.

Figure 5a represents the bi-dimensional projection (image) of the y value of the normals of the damaged painting: Figure 5b and Figure 5c are respectively the magnification of an area before and after the abrasion. It can be noticed that the morphological changes are correctly detected and there are no color variations because the abrasion of the canvas is clearly seen and represented by the normal vector images in Figure 5c.

**Figure 6.**
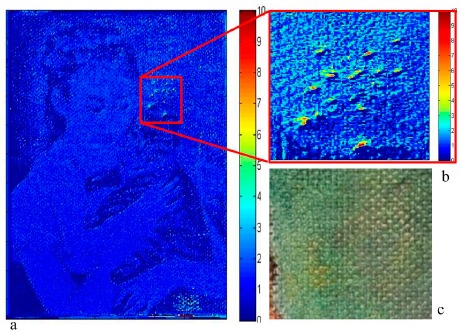
Intensity map of the changes of the damaged painting: the scale is ± σ (standard deviation) away from the average normal value of each pixel, if the normal value exceeds three the pixel can be considered changed (**a**), magnification of the intensity map of the area containing some holes (**b**) and of the color image of the damaged area (**c**).

Figure 6a represents the intensity map of the changes of the damaged painting. The map represents the distance of each pixel from the region of statistical control. The scale of the intensity map is ± σ (standard deviation) away from the average normal value of each pixel: a normal can be considered changed only if the value exceeds three times sigma.

Figure 6b represents a 10× magnification of the intensity map of the area containing some holes: the method was clearly able to detect the small holes and the intensity map of the changes was able to highlight the morphological changes caused by the holes. Figure 6c shows a 10× magnification of the color image of the damaged area: the holes are not recognizable at a human eye visual inspection.

**Figure 7.**
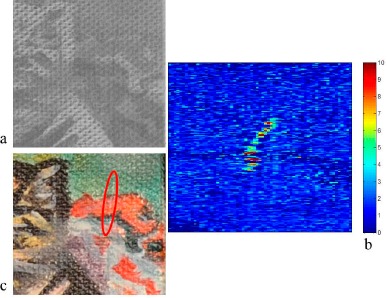
Image of the *x* values of the normals for the area containing an artificial crack (**a**), 2X magnification of the intensity map of the crack where the scale is ± σ (standard deviation) away from the average normal value of each pixel (**b**) and of the color image of the damaged area (**c**).

Figure 7a represents the bi-dimensional projection of the *x* values of the normals of the area damaged with an artificial crack: the damage is clearly recognizable and the intensity map of the changes is able to highlight the changes (Figure 7b).

The resolution of the images is 254 dpi and the width of the crack is approximately 2–3 pixels: this means that the method is able to detect morphological changes slightly smaller than 0.3 mm, which is a good result.

The painting was further damaged by creating a small dimple from the back in order to produce visually un-detectable damage (without color changes): then the RTI was captured again and the map of the changes was built.

**Figure 8.**
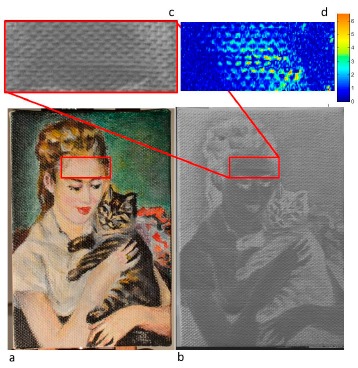
Color image of the painting after the damage caused by a dimple from the back (**a**), image of the *y* values of the normals of the damaged painting (**b**) 2× magnification of the area interested by the bend (**c**), and a 4× magnification of the intensity map of the changes of the damaged area (**d**).

In this case, the bend would be very difficult to detect visually, since it does not expose any new surface or pigment and is quite small in height. The heat map in Figure 8d shows what one expects for a bump in the surface; around it, the heat map is blue, showing little change since it far away from the bump. As one goes more to the center, there are more changes and the map turns yellow and green. Note, however, that they are still lots of blue, unchanged, pixels, even in the center of bump. This is due to fact that at the apex of the bump, the normals are roughly where they were before, in the same direction as the rest of the substrate, assuming relatively flat over the field of view.

Figure 8 represents the color images: (a) of the damaged area (red rectangle), the bi-dimensional projection of the *y* values of the normals (b) with a magnification of the damaged area (c): the damage is identified and highlighted in the intensity map of the changes (Figure 8d).

## 5. Conclusions

In this paper a reliable quantitative RTI technique was introduced for the non-invasive documentation of morphological changes in paintings. RTI provided detailed information on the geometry and morphology of the painting surface. A custom semi arch arm with LED lights to illuminate the surface of the painting was used in order to have a reproducible and quantitative method for measuring morphological changes of objects.

The method presented here was able to detect both morphological changes (holes, bends, cracks,…) and color changes (abrasion of the canvas). It was estimated that this experiment can detect physical changes slightly smaller than 0.3 mm (2–3 pixels). The sensitivity of the method can be increased by using a more sensitive camera as the camera performance is directly connected with the resolution of the methodology.

This is the first attempt to use the method as tool for monitoring the conservation of painting, and the results seems to be promising for the documentation and quantification of damages. The technique is non-invasive: a system for the automatic and fast acquisition and elaboration of the data will be developed in future. Moreover, in order to increase the accuracy and sensitivity of the method, a wider range of registration algorithms and the circulator statistics will be considered.

The method is a completely non-invasive monitoring and documentation technique: a quantitative evaluation of several types of artificial damage to the painting was obtained. This non-invasive tool could be very useful to examine paintings and artwork before they travel on loan or before and after a conservation treatment.
